# Topological Abnormalities of Pallido-Thalamo-Cortical Circuit in Functional Brain Network of Patients With Nonchemotherapy With Non-small Cell Lung Cancer

**DOI:** 10.3389/fneur.2022.821470

**Published:** 2022-02-08

**Authors:** Siwen Liu, Na Yin, Chenchen Li, Xiaoyou Li, Jie Ni, Xuan Pan, Rong Ma, Jianzhong Wu, Jifeng Feng, Bo Shen

**Affiliations:** ^1^Research Center for Clinical Oncology, Jiangsu Cancer Hospital, Jiangsu Institute of Cancer Research, The Affiliated Cancer Hospital of Nanjing Medical University, Nanjing, China; ^2^Department of Radiology, Jiangsu Cancer Hospital, Jiangsu Institute of Cancer Research, The Affiliated Cancer Hospital of Nanjing Medical University, Nanjing, China; ^3^Department of Oncology, Jiangsu Cancer Hospital, Jiangsu Institute of Cancer Research, The Affiliated Cancer Hospital of Nanjing Medical University, Nanjing, China

**Keywords:** non-small cell lung cancer, resting-state, functional MRI, graph theoretical analysis, functional brain network

## Abstract

**Introduction:**

Some previous studies in patients with lung cancer have mainly focused on exploring the cognitive dysfunction and deficits of brain function associated with chemotherapy. However, little is known about functional brain alterations that might occur prior to chemotherapy. Therefore, this study aimed to evaluate brain functional changes in patients with nonchemotherapy before chemotherapy with non-small cell lung cancer (NSCLC).

**Methods:**

Resting-state functional MRI data of 35 patients with NSCLC and 46 matched healthy controls (HCs) were acquired to construct functional brain networks. Graph theoretical analysis was then applied to investigate the differences of the network and nodal measures between groups. Finally, the receiver operating characteristic (ROC) curve analysis was performed to distinguish between NSCLC and HC.

**Results:**

Decreased nodal strength was found in the left inferior frontal gyrus (opercular part), inferior frontal gyrus (triangular part), inferior occipital gyrus, and right inferior frontal gyrus (triangular part) of patients with NSCLC while increased nodal strength was found in the right pallidum and thalamus. NSCLC also showed decreased nodal betweenness in the right superior occipital gyrus. Different hub regions distribution was found between groups, however, no hub regions showed group differences in the nodal measures. Furthermore, the ROC curve analysis showed good performance in distinguishing NSCLC from HC.

**Conclusion:**

These results suggested that topological abnormalities of pallido-thalamo-cortical circuit in functional brain network might be related to NSCLC prior to chemotherapy, which provided new insights concerning the pathophysiological mechanisms of NSCLC and could serve as promising biological markers for the identification of patients with NSCLC.

## Introduction

Lung cancer is one of the most malignant cancers with high morbidity and mortality rate for both men and women in the world. Non-small cell lung cancer (NSCLC) is one subtype of lung cancer, which accounts for 85% of all the lung cancers (1). Although lung cancer has not been associated historically with abnormalities of the central neural system, an increasing body of evidence suggests that patients with lung cancer may be associated with neurocognitive impairments (2–5). Survivors of lung cancer have a high incidence of cognitive deficits, such as executive function and working memory (6–8). The above problems may be caused by chemotherapy or by altered brain functional activity associated with cancer (9–11). However, the underlying mechanisms are not completely clear.

Advanced neuroimaging studies in patients with cancer provide a better understanding of chemotherapy-induced neurocognitive dysfunction or that related to cancer itself (12–14). Resting-state functional MRI (rs-fMRI) is a noninvasive neuroimaging technology with relatively high spatial and temporal resolution (15, 16). The method of rs-fMRI has been widely used in the studies of brain function by evaluating the temporal correlations of neuronal activity based on blood oxygenation level-dependent (BOLD) signals between different areas of the brain during rest (17–20). In addition, it has been widely implemented to explore the characteristics of functional brain networks and investigate the neuronal pathophysiology in many neuropsychiatric diseases (21, 22). Previous neuroimaging studies have shown that cross-sectional alterations of brain function were associated with cancer status while longitudinal abnormalities of brain function were related to the neurotoxic brain injury of chemotherapy (23, 24). Changes of brain function have been found to be associated with chemotherapy in patients with lung cancer in previous fMRI studies (11, 25).

Recently, graph theoretical analysis has been applied to explore the topological properties of brain networks in individuals with cognitive impairments, such as Alzheimer's disease and schizophrenia (26, 27). Using this method, the whole brain is considered as a network of nodes and edges (connections between nodes) resembling functional connections between all regions in the brain (28–31). Based on the method of graph theoretical analysis, both the changes of network measures and alterations only affecting distinct nodes (brain regions; nodal measures) can be explored (28, 32–34). A previous study using graph theoretical analysis showed that the functional brain network became inclined toward regular network in breast cancer survivors who were treated with chemotherapy and the functional brain network became inclined toward random network in breast cancer survivors who did not receive chemotherapy treatment (24). Patients with breast cancer were also found to have altered small-world properties and lower tolerance to attacks, which was associated with cognitive impairment (35). Patients with prostate cancer exhibited altered network organization and disrupted nodal characteristics in frontal and temporal regions in a previous study using diffusion-weighted imaging and graph theory (36). Patients with gynecological cancer had significant decrease in both the local efficiency and global efficiency of functional brain networks (37). However, it is unclear whether abnormal brain function already occurs in patients with lung cancer before the start of chemotherapy treatment.

The study about structural brain network demonstrated that patients with NSCLC had reduced clustering coefficient in the left hippocampus and increased shortest path length in the left middle frontal gyrus (orbital part), superior temporal gyrus and right Rolandic operculum, rectus gyrus, putamen, as well as decreased global efficacy in the left inferior frontal gyrus (triangular part), left inferior frontal gyrus (orbital part), right rolandic operculum, right gyrus rectus, right lenticular nucleus (putamen), left superior temporal gyrus and right inferior temporal gyrus and decreased local efficacy in the left middle frontal gyrus (orbital part), left superior temporal gyrus (2, 3). The changes of brain function may have preceded structural abnormalities. However, no study has examined the topological organization of the brain functional network in patients with NSCLC prior to chemotherapy.

In our clinical work, we found that many patients with lung cancer already had serious emotional and cognitive impairments before chemotherapy. However, many previous studies have ignored this problem or failed to explore the mechanisms of cancer-induced damages in the central nervous system from the perspective of neural circuits or brain networks. To better understand the impacts of chemotherapy on the brain, it is appropriate to first observe whether cancer itself has an impact on the function of the brain. Therefore, in this study, we investigated the network and nodal measures of the whole-brain functional network in patients with NSCLC who did not undergo chemotherapy. This allowed us to explore how the functional brain network was affected by the disease of NSCLC. We hypothesized that the topological properties of the functional brain network were disrupted in patients with NSCLC before chemotherapy. In addition, these altered measures might serve as potential biomarkers for distinguishing NSCLC from healthy control (HC).

## Materials and Methods

### Participants

We recruited 35 patients with NSCLC who had been diagnosed recently at the Department of Oncology, Jiangsu Cancer Hospital and Jiangsu Institute of Cancer Research and The Affiliated Cancer Hospital of Nanjing Medical University. A total of 46 age-, gender-, and education-matched HC were recruited through local advertisement. All the subjects were right-handed (self-reported) (Table 1).

**Table 1:** Demographic and clinical characteristics of participants.

**Variables**	**NSCLC (*n* = 35)**	**HC (*n* = 46)**	***t*/χ^2^**	***P***
**Age (years)**	59.69 ± 4.88	59.59 ± 4.51	0.094	0.93^a^
**Education level (years)**	13.60 ± 2.35	14.09 ± 1.53	−1.13	0.26^a^
**Gender (male/female)**	28/7	34/12	0.41	0.52^b^
**Histological diagnosis (** ***n*** **(%))**				
NSCLC	35(100%)	-	-	-
*Adenocarcinoma*	30 (86%)	-	-	-
*Squamous cell carcinoma*	4 (11%)	-	-	-
*Non-classified*	1 (3%)	-	-	-
SCLC	0(0%)	-	-	-
**Tumor stage**				
I	0 (0%)	-	-	-
IIA	0 (0%)	-	-	-
IIB	0 (0%)	-	-	-
IIIA	0 (0%)	-	-	-
IIIB	6 (17%)	-	-	-
IV	29 (83%)	-	-	-
**Maximum tumor size (cm)**				
<3	10 (28%)	-	-	-
3–5	8 (23%)	-	-	-
5–7	9 (26 %)	-	-	-
>7	8 (23%)	-	-	-
**Extracranial metastasis**				
Lung	13 (37%)	-	-	-
Bone	14 (40%)	-	-	-
Liver	6 (17%)	-	-	-
Adrenal gland	2 (6%)	-	-	-
**EGFR mutation**				
19 deletion	9 (26%)	-	-	-
T790M mutations	2 (6%)	-	-	-
L858R substitution	8 (23%)			
No mutation	16 (45%)			

*NSCLC, non-small cell lung cancer; SCLC, small cell lung cancer; HC, healthy control; EGFR, epidermal growth factor receptor. p < 0.05 was considered to be statistically significant*.

a *p-values were obtained using the two-sample t-tests*.

b *p-value was obtained using the Pearson's chi-squared test*.

Participants were eligible if they: (1) were aged from 40 to 70 years; (2) had no history of diagnosed neurological disorder, major psychiatric disorder, severe or chronic concomitant systemic illness potentially influencing brain function; (3) had no history of traumatic brain injury, disturbance of consciousness; (4) had no history of alcohol or drug abuse; and (5) had no contraindications to undergo MRI. All the patients had a histologically proven diagnosis of NSCLC and had received no radiation and/or chemotherapy treatment. Patients were excluded, if they had evidence of brain metastases on structural MRI.

This study was approved by the Medical Ethics Committee of Jiangsu Cancer Hospital and Jiangsu Institute of Cancer Research and The Affiliated Cancer Hospital of Nanjing Medical University. All the participants provided a written informed consent before this study.

### Magnetic Resonance Imaging Data Acquisition

All the MRI data were obtained with a 3.0 T Philips Aachieva Scanner. T1-weighted images were acquired with the following parameters: repetition time (TR)/echo time (TE) = 9/2.48 ms; flip angle (FA) = 9°; field of view (FOV) = 200 × 200 mm^2^; matrix = 200 × 200; voxel size = 1 × 1 × 1 mm; slice gap = 0; slice number = 176; acquisition time = 4 min 24 s. The rs-fMRI data were acquired with the following parameters: TR/TE = 3,000/40 ms; FA = 90°; FOV = 240 × 240 mm^2^; matrix = 80 × 80; voxel size = 3 × 3 × 4 mm; slice gap = 0; slice number = 34; acquisition time = 6 min 48 s.

### Magnetic Resonance Imaging Data Preprocessing

**Figure 1:**
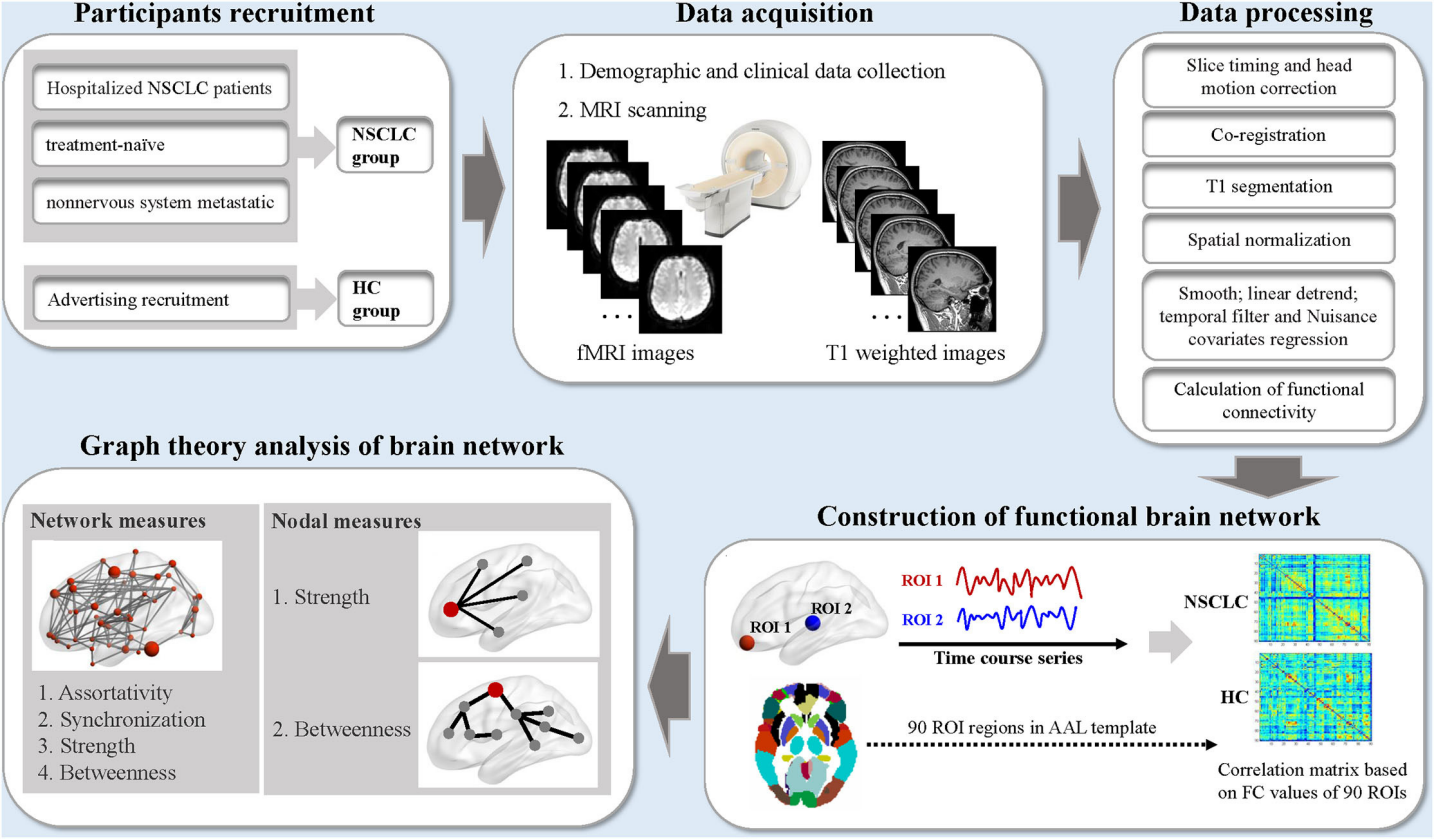
The schematic diagram for the acquisition, preprocessing, and graph theoretical analysis of resting-state functional MRI data in this study. NSCLC, non-small cell lung cancer; HC, healthy control; fMRI, functional MRI; ROI, region of interest; AAL, anatomic automatic labeling. MRI data were processed by the Data Processing Assistant for Resting State fMRI software (DPARSF). The nodal and network measures of the functional brain network were calculated by using the GRaph thEoreTical Network Analysis (GRETNA) toolbox.

Magnetic resonance imaging data were preprocessed using the Data Processing Assistant for rs-fMRI advanced edition (DPARSF) based on MATLAB and SPM (38) (Figure 1). The standard preprocessing steps were as follows: (1) the first 6 volumes were discarded for signal stabilization; (2) time differences in slice acquisition and head motion were corrected; (3) realignment to the middle image; (4) spatial normalization to the Montreal Neurological Institute (MNI) template; (5) resampled to 3 × 3 × 3 mm^3^; (6) a 4-mm full-width half-maximum (FWHM) Gaussian kernel for spatial smoothing is applied; (7) signal linear detrending was performed; (8) filtered with a temporal band-path of 0.01–0.1 Hz to reduce the low-frequency drift and high-frequency respiratory and cardiac noise; (9) nuisance covariates including the white matter signal, cerebrospinal fluid signal, and Friston 24 motion parameters were regressed out. Participants were excluded if the head motion exceeded 2 mm of translation or 2° of rotation in any direction.

### Construction of Functional Brain Network

A network can be constructed by defining nodes and estimating edges (28). The whole brain was firstly parcellated into 90 regions of interest (ROIs) by the Automated Anatomical Labeling (AAL) atlas and each of the ROI was considered as a node in the functional brain network (39). *Pearson's* correlations between the time series of all ROIs were extracted and were transformed into z-scores via *Fisher's* transformation, which were defined as the connection strength of edges in the functional brain network. Finally, a functional connectivity matrix was obtained, and then undirected weighted network was constructed.

### Calculation of Network and Nodal Measures

The network and nodal measures of the functional brain network were calculated using the GRETNA toolbox (40). For the functional brain network at each sparsity threshold, we calculated the following parameters: (1) network and nodal measures including assortativity, synchronization, strength, and betweenness; (2) nodal measures including strength and betweenness (28).

Nodal strength is computed as the sum of weights of all edges that are connected to it in the whole network (41). Nodal betweenness is calculated as the number of all shortest paths that pass through it in the while network (42). Network assortativity is considered as the nodal strength correlation between connected nodes in the network (43, 44). A network with high assortativity indicates that nodes with similar nodal strength tend to be connected to each other (45, 46). In this study, assortativity reflects the tendency of brain regions to be connected to other regions with the same or similar nodal strength. Network synchronization measures how likely it is that all nodes fluctuate in the same wave pattern (47). More detailed information about these measures could be found in the previous study (28). To investigate the group differences of the functional brain network, area under the curve (AUC) was calculated over sparsity ranges for each measure. The AUC metric is a summarized scalar, which is free from single threshold selection and is sensitive at detecting topological characteristics of the brain network. In addition, nodes were identified as hub regions with nodal strength and betweenness at least one SD greater than the mean of all the nodes.

### Statistical Analysis

Comparisons of both the network and nodal measures between patients with NSCLC and HC were performed by using the independent two-sample two-tailed *t*-tests. In addition, the false discovery rate (FDR) was used for multiple comparison corrections (48). The FDR-corrected significance level was set at *P* < 0.05. Furthermore, the receiver operating characteristic (ROC) curves were generated to investigate the discriminating values of both network and nodal measures for discriminating patients with NSCLC from HC. *P* < 0.05 was considered as statistically significant.

## Results

### Differences of Network Measures Between the Groups

**Figure 2:**
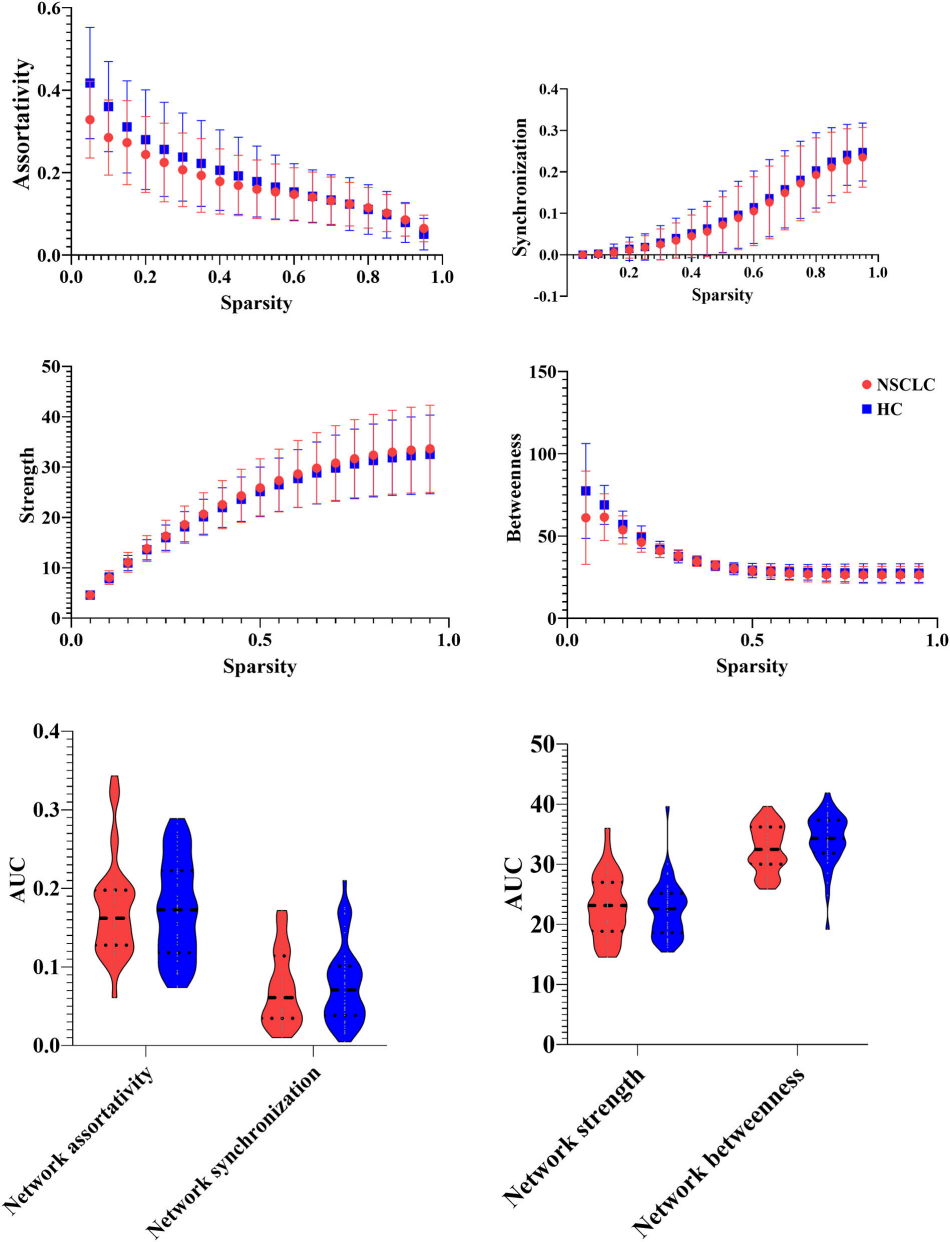
Group differences in the network measures of the functional brain networks among different sparsity thresholds (0.05–0.95) between the groups.

In this study, the functional brain networks of all the participants were thresholded with varied sparsity from 0.05 to 0.95 with step 0.05. No significant differences were found in the assortativity and synchronization of the functional brain network between NSCLC and HC (sparsity ranges: 0.05–0.95). In addition, there were no significant group differences in the strength and betweenness of the functional brain network across the sparsity range from 0.05 to 0.95. Moreover, patients with NSCLC exhibited no significant differences in the AUC of these network measures when compared with HC (sparsity ranges from 0.05 to 0.95) (Figure 2).

### Differences of Nodal Measures Between the Groups

**Figure 3:**
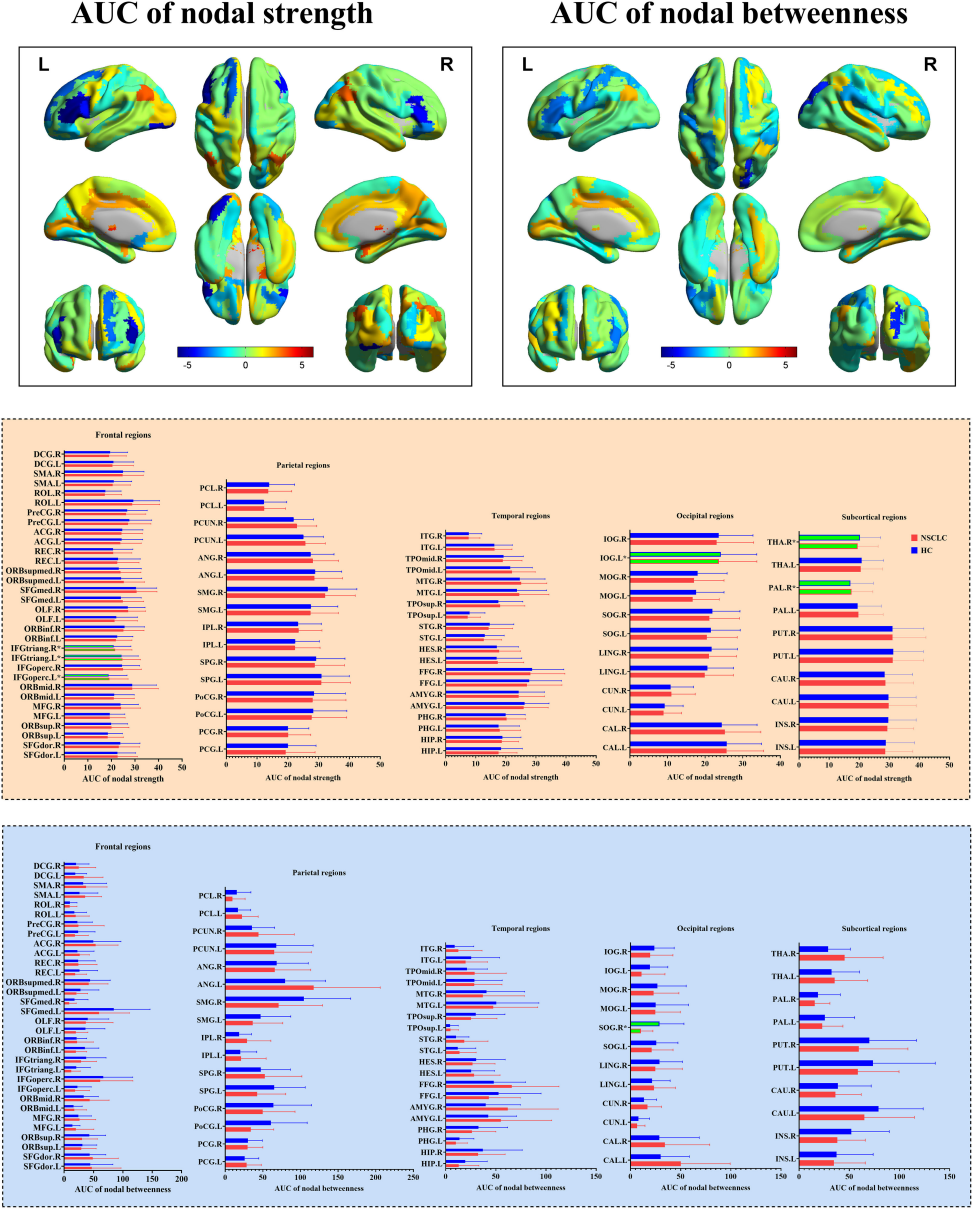
Group differences in the nodal measures of the functional brain networks between NSCLC and HC. NSCLC, non-small cell lung cancer; HC, healthy control. The superscript * and green column indicated brain regions with significant differences surviving false discovery rate (FDR) correction. The color bar indicated *t-*values of the two-sample *t*-tests. The abbreviations of brain regions were presented in the Supplementary Material online.

In the range of 0.05–0.95, the AUC of the nodal strength and betweenness in the functional brain networks for the NSCLC and HC groups were calculated and then compared. The decreased AUC of the nodal strength was found in the left inferior frontal gyrus (opercular part), inferior frontal gyrus (triangular part), inferior occipital gyrus, and right inferior frontal gyrus (triangular part) of patients with NSCLC when compared with HC while increased AUC of the nodal strength was found in the right pallidum and thalamus. Furthermore, patients with NSCLC showed decreased AUC of the nodal betweenness in the right superior occipital gyrus when compared with HC. All the above differences survived FDR correction (Table 2; Figure 3).

**Table 2:** Brain regions showed altered nodal measures in the functional brain networks of patients with NSCLC.

**Brain regions**	**NSCLC**	**HC**	***t***	***P***
**Nodal strength**				
Left inferior frontal gyrus (opercular part)	11.55 ± 6.93	18.85 ± 7.77	−4.38	0.000036
Left inferior frontal gyrus (triangular part)	15.35 ± 7.02	24.30 ± 7.10	−5.64	0.00000025
Right inferior frontal gyrus (triangular part)	13.60 ± 7.148	20.93 ± 7.34	−4.50	0.000023
Left inferior occipital gyrus	14.50 ± 8.03	24.21 ± 9.54	−4.85	0.000006
Right pallidum	23.38 ± 7.70	17.08 ± 7.60	3.68	0.00043
Right thalamus	27.28 ± 8.60	20.26 ± 6.88	4.08	0.00011
**Nodal betweenness**				
Right superior occipital gyrus	10.34 ± 12.00	28.90 ± 24.38	−4.50	0.000027

*NSCLC, non-small cell lung cancer; HC, healthy control. False discovery rate (FDR)-adjusted p < 0.05 was considered to be statistically significant*.

### Differences of Hub Regions Distribution Between the Groups

**Figure 4:**
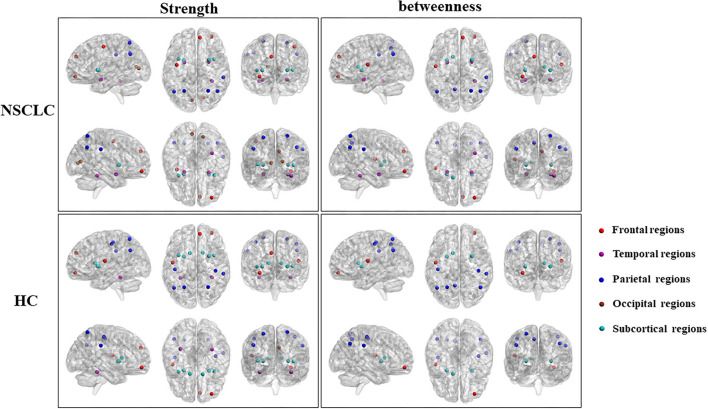
Differences in the distribution of hub regions between NSCLC and HC. NSCLC, non-small cell lung cancer; HC, healthy control. The abbreviations of brain regions were presented in the Supplementary Material online.

First, nodes with high nodal strength were classified as hub regions, which had intense interconnectivity with other regions in the brain and played an important in the information transfer and integration. Seventeen such hubs were identified in the NSCLC group, including 3 frontal regions, 3 temporal regions, 5 parietal regions, 2 occipital regions, and 4 subcortical regions. In the HC group, 17 regions were also identified as hubs, including 3 frontal regions, 2 temporal regions, 6 parietal regions, and 6 subcortical regions. Eleven of these regions (left and right superior parietal gyrus, putamen, and insula; left anterior cingulate gyrus; right middle frontal gyrus (orbital part), superior frontal gyrus (medial), fusiform gyrus, and supramarginal gyrus) were shared by both the NSCLC and HC groups. The NSCLC group lost hub properties in the left and right postcentral gyrus, caudate nucleus, left rolandic operculum, and fusiform gyrus, while the left and right amygdala, calcarine fissure, left precentral gyrus, and right anterior cingulate gyrus acted as new hub regions in the patient group (Table 3; Figure 4).

**Table 3:** Hub regions based on nodal strength and betweenness between NSCLC and HC.

**Based on strength**		**Based on betweenness**	
**NSCLC**	**HC**	**NSCLC**	**HC**
**PreCG.L** ^a^	**ROL.L** ^a^	ROL.L ^a^	ROL.L ^a^
ORBmid.R ^a^	ORBmid.R ^a^	ORBmid.R ^a^	ORBmid.R ^a^
SFGmed.R ^a^	SFGmed.R ^a^	**SFGmed.R** ^a^	**PoCG.L** ^c^
**AMYG.L** ^b^	**FFG.L** ^b^	**AMYG.L** ^b^	**PoCG.R** ^c^
**AMYG.R** ^b^	FFG.R ^b^	**AMYG.R** ^b^	ANG.L ^c^
FFG.R ^b^	**PoCG.L** ^c^	**FFG.R** ^b^	ANG.R ^c^
ANG.L ^c^	**PoCG.R** ^c^	ANG.L ^c^	PCUN.L ^c^
**ANG.R** ^c^	ANG.L ^c^	ANG.R ^c^	**SPG.L** ^c^
SPG.L ^c^	SPG.L ^c^	PCUN.L ^c^	SMG.R ^c^
SPG.R ^c^	SPG.R ^c^	**SPG.R** ^c^	CAU.L ^e^
SMG.R ^c^	SMG.R ^c^	SMG.R ^c^	PUT.L ^e^
**CAL.L** ^d^	**CAU.L** ^e^	CAU.L ^e^	PUT.R ^e^
**CAL.R** ^d^	**CAU.R** ^e^	PUT.L ^e^	
PUT.L ^e^	PUT.L ^e^	PUT.R ^e^	
PUT.R ^e^	PUT.R ^e^		
INS.L ^e^	INS.L ^e^		
INS.R ^e^	INS.R ^e^		

*NSCLC, non-small cell lung cancer; HC, healthy control*.

a *Frontal regions*.

b *Ttemporal regions*.

c *Parietal regions*.

d *Occipital regions*.

e *Subcortical regions*.

*Bold names showed different hub regions between groups. The abbreviations of brain regions were presented in the Supplementary Material online*.

Second, nodes with high nodal betweenness were classified as hub regions, which lied on the shortest path between two other regions and played an important role in controlling information flow. Based on this definition of hub regions, patients with NSCLC presented 14 brain regions (3 frontal regions, 3 temporal regions, 5 parietal regions; 23 subcortical regions) acting as hubs while HC presented 12 regions (2 frontal regions, 7 parietal regions; 3 subcortical regions). Nine of these regions (left and right anterior cingulate gyrus and putamen; left rolandic operculum, precuneus, and caudate nucleus; right middle frontal gyrus (orbital part) and supramarginal gyrus) were shared by two groups. The left and right amygdala, right superior frontal gyrus (medial), fusiform gyrus and superior parietal gyrus were defined as hub regions in the NSCLC group, but not in the HC group. In the left and right postcentral gyrus, left superior parietal gyrus was defined as hub regions only in HC (Table 3; Figure 4).

### Receiver Operating Characteristic for Discrimination Between NSCLC and HC

**Figure 5:**
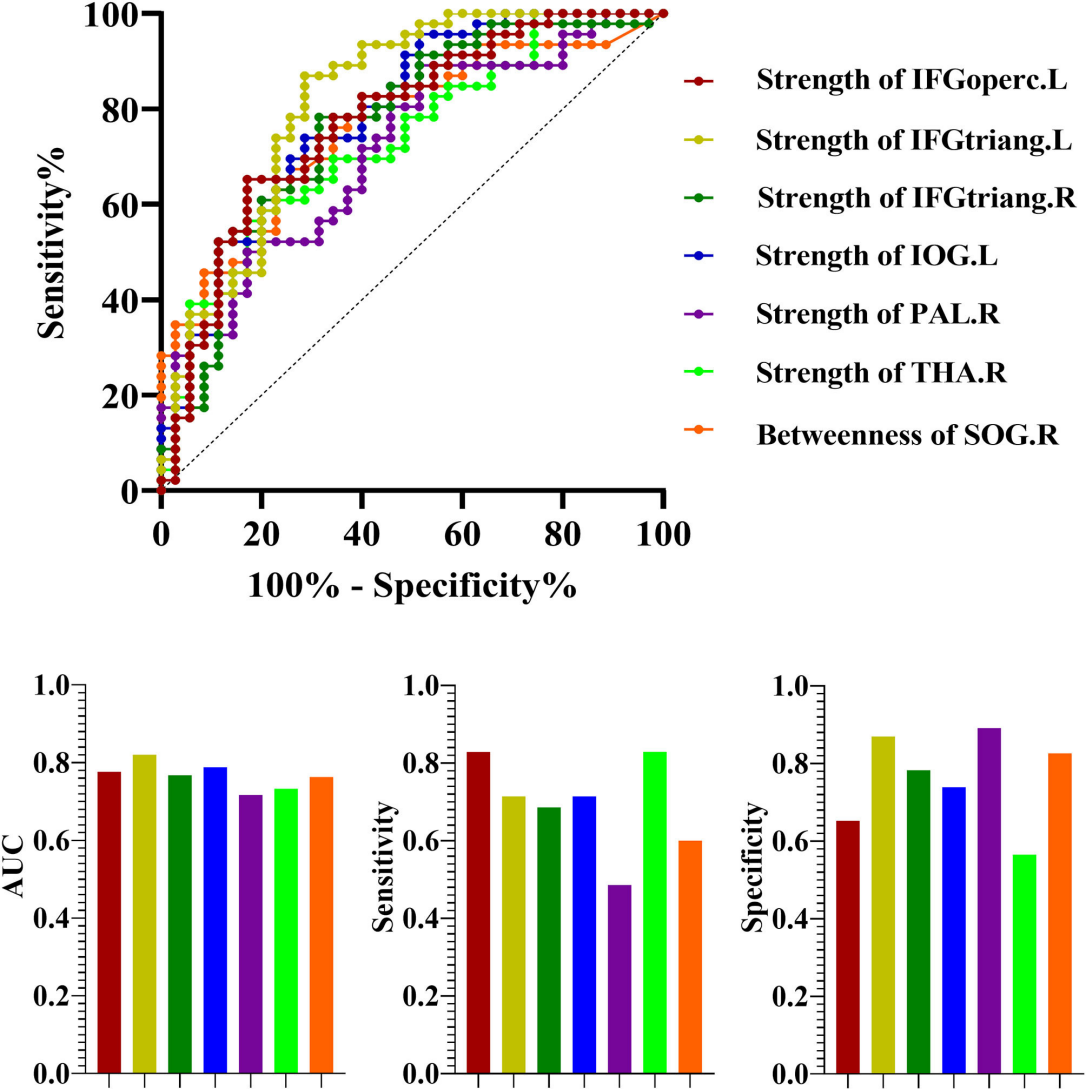
The receiver operating characteristic curve (ROC) for discrimination between NSCLC and HC. NSCLC, non-small cell lung cancer; HC, healthy control; AUC, area under the curve. The abbreviations of brain regions were presented in the Supplementary Material online.

Significant differences of the nodal measures suggested that they could be useful biomarkers for discriminating NSCLC from HC. ROC curves were generated and AUCs were calculated (Figure 5). The ROC analysis demonstrated that the strength of left inferior frontal gyrus (opercular part) (AUC = 0.78; *p* < 0.001, 95% CI: 0.67–0.88; sensitivity: 82.86% and specificity: 65.22%), inferior frontal gyrus (triangular part) (AUC = 0.82; *p* < 0.001, 95% CI: 0.72–0.92; sensitivity: 71.4% and specificity: 86.96%), inferior occipital gyrus (AUC = 0.79; *p* < 0.001, 95% CI: 0.69–0.89; sensitivity: 71.4% and specificity: 73.91%) and right inferior frontal gyrus (triangular part) (AUC = 0.77; *p* < 0.001, 95% CI: 0.66–0.87; sensitivity: 68.6% and specificity: 78.26%), pallidum (AUC = 0.72; *p* < 0.001, 95% CI: 0.61–0.83; sensitivity: 48.6% and specificity: 89.13%) and thalamus (AUC = 0.73; *p* < 0.001, 95% CI: 0.62–0.84; sensitivity: 82.9% and specificity: 56.52%) exhibited good performance in distinguishing patients with NSCLC from HC, as well as the betweenness of right superior occipital gyrus (AUC = 0.76; *p* < 0.001, 95% CI: 0.66–0.87; sensitivity: 60% and specificity: 82.61%).

## Discussion

In this study, there were no significant differences in the network assortative and synchronization between patients with NSCLC prior to chemotherapy and HC. However, significant group differences were found in the nodal strength and betweenness, mainly located in the frontal and subcortical regions. The frontal regions showed decreased nodal strength while increased nodal strength was identified in the pallidum and thalamus of patients with NSCLC. These findings demonstrated that abnormal topological features of the pallido-thalamo-cortical circuit might be associated with NSCLC, which occurred in the functional brain network of patients with NSCLC before the start of chemotherapy treatment. In addition, the ROC curve revealed that the abnormal nodal measures of pallido-thalamo-cortical circuit exhibited robust accuracy and excellent specificity in distinguishing NSCLC from HC. Therefore, it appeared that nodal strength and betweenness of these brain regions could be used to characterize the severity of NSCLC, at least to some extent.

Chemotherapy-related cognitive impairment (chemobrain) had been found in patients with cancer outside the central nervous system in previous neuropsychological studies (49, 50). Chemotherapy-related cognitive impairment was explored from 1978, however, the first study about cancer-related cognitive impairment was conducted in 2008 (51, 52). The cognitive impairment was detected in 20–30% of patients with cancer outside the central nervous system before the start of chemotherapy (53, 54). Therefore, cancer itself might induce biological mechanisms, which are involved in the development of cognitive impairment in patients with cancer. The brain had been found to play an essential role in the cognitive function, such as attention, memory, and executive function (55, 56). With the development of neuroimaging methods, especially fMRI, the relationships between brain activity and cognition could be revealed (57, 58). Previous fMRI studies had found that many cognitive processes were located in the frontal regions of the brain, which could be activated by tasks related to cognition (59, 60). However, the underlying neurobiological mechanisms related to cancer-related cognitive impairment had not been elucidated.

In this study, increased nodal strength was found in the left inferior frontal gyrus (opercular part), inferior frontal gyrus (triangular part), inferior occipital gyrus, and right inferior frontal gyrus (triangular part) of patients with NSCLC. The inferior frontal gyrus was found to be related to the process such as attention and executive function and non-invasive brain stimulation of the inferior frontal gyrus significantly improved the cognition (61). In addition, the connections between the inferior frontal gyrus and subcortical regions were found to be involved in the executive processing (62). Decreased functional connectivity between the left inferior frontal gyrus (triangular part) and dorsolateral prefrontal cortex was found in patients with lung cancer after chemotherapy when compared with HC while decreased functional connectivity between the left inferior frontal gyrus and the right dorsolateral prefrontal cortex was identified in patients with lung cancer after chemotherapy when compared with those before treatment (6). Abnormal functional connectivity between these two brain regions was found to be correlated to the worse neuropsychologic performance of patients with cancer (63). However, hyperactivity was found in the right inferior frontal gyrus and several other regions in the pathway of visceral to brain signal transduction in patients with NSCLC prior to oncotherapy by using resting-state 18F-fluoro-D-glucose (FDG) PET/CT and the abnormal glucose metabolism might be attributed to NSCLC related visceral sympathetic activation (64).

The interactions between the brain and cancer outside the central nervous system suggested that cancer information could be conveyed into the brain by the special structures within the spinal and cranial and nerves and the brain could consequently regulate the growth of cells of cancer in peripheral tissues by the neuroendocrine-immune system (65). Therefore, non-central nervous system cancer itself could induce abnormal function of certain regions in the brain, which might play an essential role in the progression and metastases of cancer. Decreased nodal strength of bilateral inferior frontal gyrus in this study suggested that reduced functional connectivity between these regions and other regions occurred before the treatment of chemotherapy. This finding was consistent with previous studies, which indicated that patients with cancer had cognitive impairment or emotional problems prior to chemotherapy, as well as impaired brain function or structure. The decreased functional connectivity of the inferior frontal gyrus might also lead to the reduced level of executive control and increased speed of cancer growth.

Meantime, the nodal strength of the right pallidum and thalamus was increased in the NSCLC group. These two subcortical regions were the central components of the cancer-to-brain pathway, which might be hyperactivated as a compensatory reaction (65). The pallidum and thalamus are strongly connected with the cortical regions, especially pallido-thalamo-cortical circuit, which is involved in a variety of cognitive processes (66, 67). Abnormal activation of pallidum was identified in patients with cancer, which might lead to impaired reward and motivation processing (68). Increased activation during working memory was found in the right thalamus in patients with cancer before chemotherapy (69). In addition, the cognitive impairment together with structural brain changes in the thalamus was found in patients with lung cancer (70). The pallidum, one component of the basal ganglia, was found to be associated with the cognitive performance of patients (71). The thalamus had complex connections with the prefrontal regions and thalamo-cortical connections had been found to be involved in various cognitive processes (72). Therefore, we suspected that the increased functional connectivity of the pallidum and thalamus in this study might reflect an attempt to reinstate homeostasis in functions related to cancer and the visceral sympathetic activation.

No significant group differences were found in the network assortative and synchronization. An assortative network has highly connected nodes, which improves network resilience and reduces disruption loss (73, 74). Therefore, this type of network facilitates the spread of information over the network (75). The index of network synchronization reflects the possibility of all the nodes fluctuate in the same wave pattern (47, 76). In this study, there were no significant changes in the global measures including network assortative and synchronization, which suggested that NSCLC was not enough to cause topological properties changes of the functional brain network from the global perspective. Based on both nodal strength and betweenness, patients with NSCLC lost hub properties in some regions, while some hub regions were identified in patients with NSCLC, which might represent a compensatory mechanism. Highly connected nodes or nodes with high betweenness are defined as hubs, which play a key role in the integration of information (28, 77). In this study, no selective damages were found in these hub regions, therefore, the global characteristics of the functional brain networks were not impaired in patients with NSCLC.

These findings about cancer-induced impacts on the brain are of great significance to explore whether brain function or microstructure damages caused by non-central nervous system metastatic cancers can predict the probability or mechanisms of brain metastasis in advanced cancers in the future studies. Although altered nodal measures could be useful biomarkers for discriminating NSCLC from HC by the method of the AUC, we would explore the validity of the prediction with different machine learning models with the increase of sample size and longitudinal follow-up data. In addition, although rs-fMRI is a valid method to observe the changes of brain function and microstructure, this method may be indirect when compared with other methods, such as animal experiments. Therefore, we will also establish NSCLC animal rat models, and observe the cognitive and emotional impairments of rats before and following the use of chemotherapeutic drugs longitudinally in our future studies. The level of dopamine (DA) and 5-hydroxytryptamine (5-HT) in the related brain areas will also be compared before and following chemotherapy. Finally, we aim to provide the theoretical basis for optimizing the efficacy of NSCLC chemotherapy and reducing treatment-related cognitive and emotional impairments, and further to develop the individualized and precise treatments for NSCLC.

Several limitations of this study should be further discussed. First, the sample size was relatively small, which might reduce the generalizability of our results to some extent. Second, the present study was a cross-sectional study, which could not elucidate the causal links between NSCLC and abnormal functional connectivity. Third, lacking the results of cognitive performance did not allow us to explore the relationships between altered brain function and cognitive impairment of patients with NSCLC. Finally, smoking and lifestyle might be also associated with the development of NSCLC and these factors should be considered in our further task-based fMRI studies.

## Conclusion

In conclusion, this was the first study to explore the topological features of the functional brain networks of patients with NSCLC prior to chemotherapy. Despite the limitations of this study, the findings revealed that abnormal functional connectivity of the pallido-thalamo-cortical circuit occurred before the start of chemotherapy treatment, which also showed good performance in distinguishing NSCLC from HC. Therefore, this circuit might be a potential biomarker for NSCLC from the perspective of cancer.

## Data Availability Statement

The original contributions presented in the study are included in the article/Supplementary Material, further inquiries can be directed to the corresponding authors.

## Ethics Statement

The studies involving human participants were reviewed and approved by the Medical Ethics Committee of Jiangsu Cancer Hospital and Jiangsu Institute of Cancer Research and The Affiliated Cancer Hospital of Nanjing Medical University. The patients/participants provided their written informed consent to participate in this study.

## Author Contributions

SL, JF, BS, and JW designed the experiments. SL, NY, CL, XL, JN, XP, and RM contributed to clinical data collection and assessment. SL, NY, CL, and XL analyzed the results and contributed to the manuscript. SL, JF, and BS approved the final manuscript. All authors contributed to the article and approved the submitted version.

## Funding

This study was supported by the grants of the National Natural Science Foundation of China (No. 81871873), the Foundation of Jiangsu Cancer Hospital (No. ZM201923), the Jiangsu Provincial Top Talent Scientific Research Project of Six One Projects for High Level Health Talents (No. LGY2019076), and the Young Talents Program of Jiangsu Cancer Hospital (No. QL201814).

## Conflict of Interest

The authors declare that the research was conducted in the absence of any commercial or financial relationships that could be construed as a potential conflict of interest.

## Publisher's Note

All claims expressed in this article are solely those of the authors and do not necessarily represent those of their affiliated organizations, or those of the publisher, the editors and the reviewers. Any product that may be evaluated in this article, or claim that may be made by its manufacturer, is not guaranteed or endorsed by the publisher.
